# Supplementary figures and images for: Successional, spatial, and seasonal changes in seed rain in the Atlantic forest of southern Bahia, Brazil
Source: PLoS One. 2019 Dec 16;14(12):e0226474. doi: 10.1371/journal.pone.0226474 (PMC6913908; doi:10.1371/journal.pone.0226474)

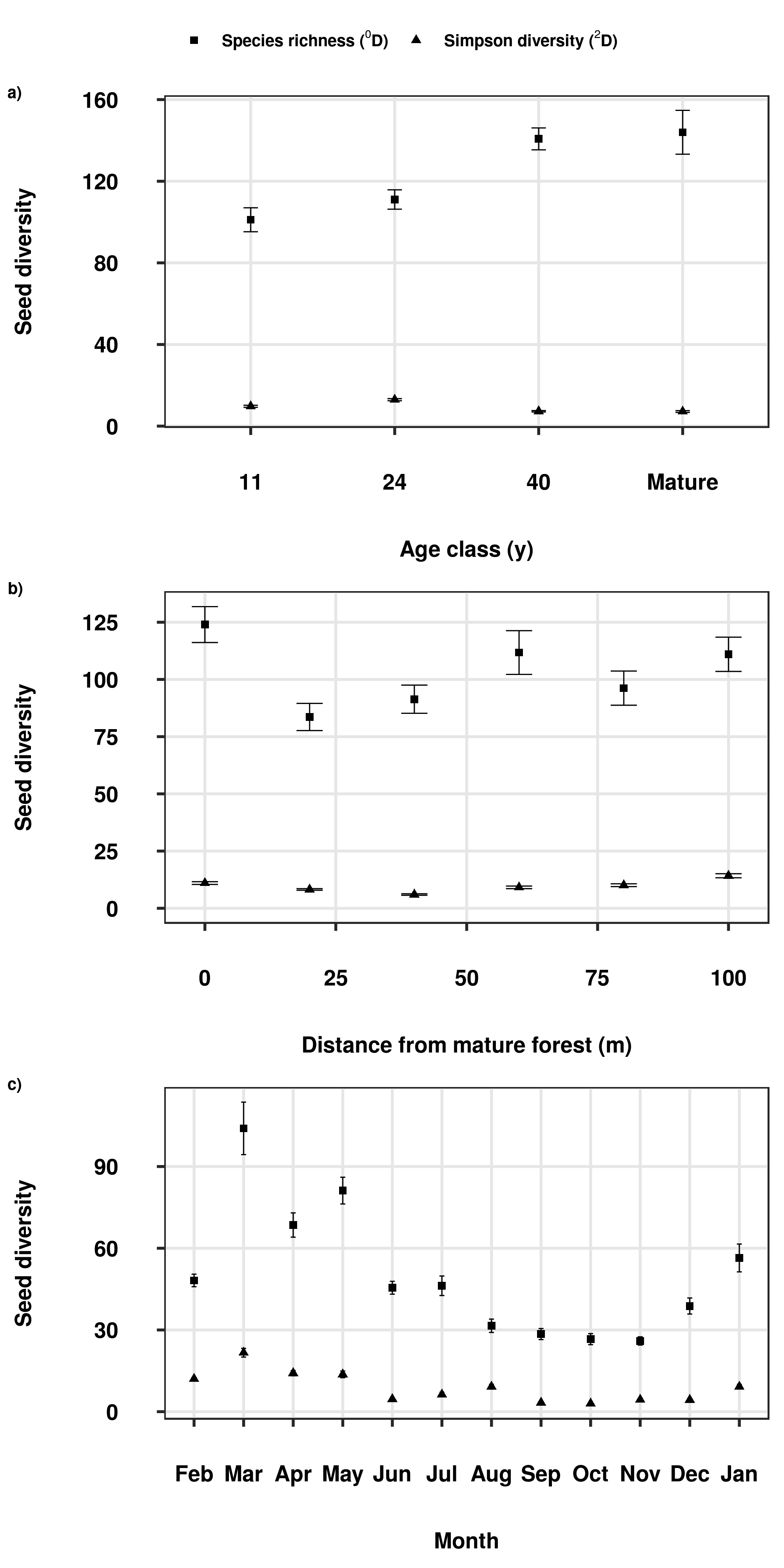

Supplement: S1 Fig — Diversity estimates were made using sample coverage-based rarefaction and extrapolation. Sample coverage was 99.3% for a), 96.5% for b), and 98.8% for c). Whisker bars are 95% confidence intervals. (TIFF) [file pone.0226474.s001.tiff]

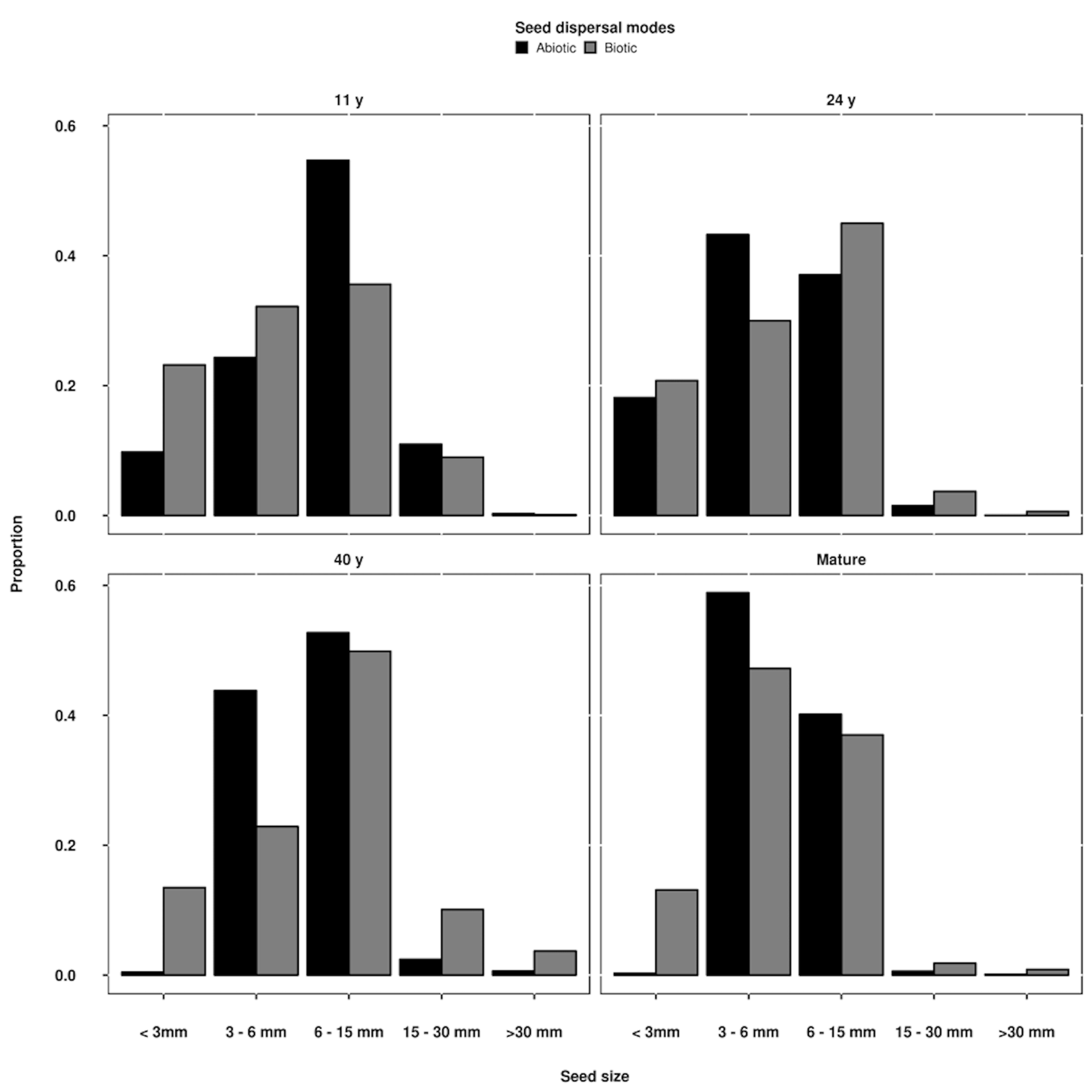

Supplement: S2 Fig — Proportion of seed class sizes was calculated by pooling one year of seed rain density across forest age classes. (TIFF) [file pone.0226474.s002.tiff]
